# PoET: automated approach for measuring pore edge tension in giant unilamellar vesicles

**DOI:** 10.1093/bioadv/vbab037

**Published:** 2021-11-23

**Authors:** Fernanda S C Leomil, Marcelo Zoccoler, Rumiana Dimova, Karin A Riske

**Affiliations:** 1 Departamento de Biofísica, Universidade Federal de São Paulo, São Paulo 043039-032, Brazil; 2 Department of Theory and Bio Systems, Max Planck Institute of Colloids and Interfaces, Potsdam 14424, Germany; 3 DFG Cluster of Excellence “Physics of Life”, Technical University of Dresden, Dresden 01307, Germany

## Abstract

**Motivation:**

A reliable characterization of the membrane pore edge tension of single giant unilamellar vesicles (GUVs) requires the measurement of micrometer sized pores in hundreds to thousands of images. When manually performed, this procedure has shown to be extremely time-consuming and to generate inconsistent results among different users and imaging systems. A user-friendly software for such analysis allowing quick processing and generation of reproducible data had not yet been reported.

**Results:**

We have developed a software (PoET) for automatic pore edge tension measurements on GUVs. The required image processing steps and the characterization of the pore dynamics are performed automatically within the software and its use allowed for a 30-fold reduction in the analysis time. We demonstrate the applicability of the software by comparing the pore edge tension of GUVs of different membrane compositions and surface charges. The approach was applied to electroporated GUVs but is applicable to other means of pore formation.

**Availability and implementation:**

The complete software is implemented in Python and available for Windows at https://dx.doi.org/10.17617/3.7h.

**Supplementary information:**

Supplementary data are available at *Bioinformatics Advances* online.

## 1 Introduction

Giant unilamellar vesicles (GUVs) have proven to be a handy model for studying membrane-involving cellular processes, because they have sizes comparable to cells (10–100 µm), can mimic plasma membrane curvature, and allow for membrane interactions and responses to external disturbances to be directly observed under an optical microscope (Dimova, 2019; Walde *et al.*, 2010). GUVs have been used in several applications to measure a multitude of membrane characteristics, such as elastic, rheological, electrical and thermodynamic properties. These include membrane bending rigidity (Döbereiner *et al.*, 2003; Faizi *et al.*, 2020), stretching elasticity and lysis (Henriksen and Ipsen, 2004; Rawicz *et al.*, 2000), diffusivity of molecules (Schwille *et al.*, 1999), viscosity (Dimova *et al.*, 1999; Honerkamp-Smith *et al.*, 2013), capacitance (Garten *et al.*, 2017; Salipante *et al.*, 2012), edge tension (Portet and Dimova, 2010) and phase separation (Baumgart *et al.*, 2003; Dietrich *et al.*, 2001), most of which were recently summarized in Dimova and Marques (2019). Among these, the pore edge tension (sometimes referred to as line tension) defines the stability of pores formed in the membrane, drives their closure, and is thus an essential characteristic for plasma membrane repair processes. The pore edge tension reflects the energy cost per unit length of maintaining a membrane pore opened. It is an intrinsic membrane characteristic arising from the physicochemical properties and the amphiphilic nature of lipid molecules in the bilayer (Brochard-Wyart *et al.*, 2000). It is intimately related to membrane stability and plays a crucial role in membrane resealing mechanisms after distinct membrane perturbations causing pore formation, such as ultrasound (Newman and Bettinger, 2007), electroporation (Escoffre *et al.*, 2009; Zhelev and Needham, 1993), light-driven poration (Sandre *et al.*, 1999), osmotic swelling Shibly, S. U. A. *et al.*, 2016) and peptide interaction (Lee *et al.*, 2008). In particular, electroporation, which is the opening of transient membrane pores in response to a high-intensity electric field, leading to an abrupt increase in membrane permeability, has become a common approach for different medical applications, such as in the treatment of different types of cancer (Belehradek *et al.*, 1993; Edhemovic *et al.*, 2011), gene therapy (Heller and Heller, 2010; Keating and Toneguzzo, 1990) and to encapsulate or promote cargo release in drug delivery systems (Alvarez-Erviti *et al.*, 2011; Perrier *et al.*, 2017). It has several applications in biotechnology (Kotnik *et al.*, 2015) and it is also frequently used for basic research purposes (Neumann *et al.*, 1982; Potter and Heller, 2018). Due to its practicality and efficiency, it has also been extensively used to investigate membrane properties in model systems (Dimova *et al.*, 2007, 2009; Karal *et al.*, 2019; Muralidharan *et al.*, 2021; Perrier, 2018; Riske and Dimova, 2005). All these applications, and, in principle, the interest in understanding membrane poration in vesicles motivate the quest for a fast and easy approach of evaluating the edge tension of membranes.

**Fig. 1.**
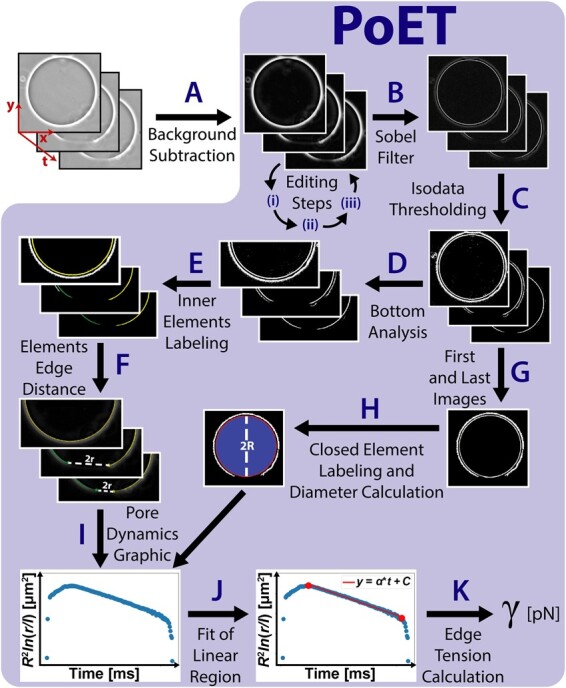
PoET method flowchart. The individual steps are explained in detail in the text.

In a previous work, Portet and Dimova (2010) proposed a method for measuring pore edge tension in GUVs subjected to DC electric pulses. This method is based on the theoretical work developed by Brochard-Wyart *et al.* (2000) that describes pore dynamics in a spherical vesicle as a four-stage process: (i) pore growth, (ii) pore stabilization at a maximum radius, (iii) slow reduction of the pore size and (iv) fast closure (see examples given in Figs. 1I and J and 3B). A major part of the pore lifetime is comprised in the third stage—the slow leak-out regime, which is linear and used to determine the edge tension. Recordings of the time dependence of a closing pore allow us to assess the edge tension γ by means of the following equation:

(1) $$R^{2}\ln\left(r\right)=-\left(\frac{2\gamma }{3\pi \eta }\right)t+C,$$

where R is the GUV radius after pore closure, r is the pore radius over time t, η is the viscosity of the aqueous medium and *C* is a constant dependent on each experiment. This equation is valid under the assumptions that the membrane is incompressible, the membrane tension is uniform and the vesicle radius does not vary significantly during the pore closure stage [for details on the model and the various contributions at the different stages of pore opening and closure, see Brochard-Wyart *et al.* (2000) and Ryham *et al.* (2011)]. Thus, in order to measure the pore edge tension, one has to record the vesicle, resolve the pore over time and using Equation (1) obtain γ from the pore closure time dependence. A typical experiment for measuring the pore edge tension involves the application of a DC electric pulse on a selected GUV. In the presence of an electric field, free charges in the inner and outer vesicle solution accumulate across the membrane, which acts as a capacitor. The characteristic charging time of this capacitor depends on the conductivities of the inner and outer solutions, the vesicle diameter and the membrane capacitance (Chiabrera *et al.*, 1985; Riske and Dimova, 2005). For high ionic strength solution, the charging time is very low but can be as high as 500 µs for a solution with minor amounts of salt and a typical GUV diameter. The electric field builds up a transmembrane potential across the membrane (Kinosita *et al.*, 1988) up to a threshold value known as critical electroporation potential, above which the probability of membrane rupture increases considerably and transient pores appear, rendering the membrane permeable to ions. The electric DC pulses applied are frequently chosen to be square-wave pulses and the internal and external aqueous solutions are usually sucrose and glucose, respectively, in order to ensure osmotic and gravity stabilization as well as to guarantee a clear vesicle contour under contrast-enhancing modes of observation. When sucrose-loaded GUVs placed in a glucose external solution are observed by phase-contrast microscopy, an intensity line profile across their equator displays a sharp dark to bright transition at the GUV membrane from the inside to the outside—which constitutes the interface of two different refractive index solutions—followed by a typical bright halo inherent of phase-contrast imaging, see Figure 1A. Once a pore opens, the local mixing of the solutions dissipates the intensity gradient inside the pore, allowing its size to be measured. DC pulses applied on GUVs made of phosphatidylcholine (PC) were shown to induce opening of macropores (diameter of a few µm) that usually last about ∼50 ms (Riske and Dimova, 2005). Fast digital imaging can be used to track pore closure rates so that the edge tension can be calculated with Equation (1). This analysis reliability depends on having enough data to characterize the pore dynamics, which is limited not only by acquisition rate, but also by pore lifetime, which in turn increases with GUV size. This means that smaller GUVs tend to need higher acquisition framerates to have their pore dynamics well characterized.

When manually performed, the procedure of measuring pore closure rate (by tracking pore diameter along image sequences containing hundreds to thousands of frames) has proven to be extremely time-consuming, due to the large amount of data required for reliable characterization, and also biased, since manual measurements on digital images may be quite inconsistent among different users and imaging systems. This may be one of the reasons why edge tension values vary considerably among different research groups (Portet and Dimova, 2010). By making this procedure automatic, one can significantly accelerate experimental video analysis and can also produce more reliable and reproducible edge tension values.

Here, we present an original method, built-in as a user-friendly free and open-source software called PoET (Pore Edge Tension). PoET is simple and easy to use and the required image processing steps are executed automatically within the software. It is equipped with error-rectifying mechanisms and alerts that prevent it from crashing while guiding the user throughout a more powerful analysis. To our knowledge, this is the first software designed for edge tension measurement.

We used experimental data to validate PoET and demonstrate its applicability, by quantifying the edge tension of palmitoyl oleoyl PC GUVs containing up to 50 mol% of biologically relevant anionic lipids. In a previous study, we showed that the presence of 50 mol% of palmitoyl oleoyl phosphatidylglycerol (PG) (with one negative charge) in PC membranes can lead to vesicle collapse after electroporation (Riske *et al.*, 2009) and this effect was directly related to a substantial reduction of the edge tension (Lira *et al.*, 2021). Here, we use PoET to measure the effect of other anionic lipids on membrane edge tension, namely phosphatidylserine (PS), cardiolipin (CL), phosphatidylinositol (PI) and phosphatidylinositol 4,5-bisphosphate (PIP2) and compare them with PG. PS has one net negative charge and is found predominantly in the inner leaflet of eukaryotic cellular membranes playing a key role in cell apoptosis (Fadok *et al.*, 1998). CL, with four acyl chains and two negative charges on its polar head, is the main phospholipid found in the inner mitochondrial membrane (Bevers *et al.*, 1996). It plays an important role in the molecular organization and function of this organelle, as well as in its permeability, in addition to having its oxidation related to the activation of cell apoptosis and in the development of several diseases (Unsay *et al.*, 2013). PI and PIP2, with one and three negative charges on their polar heads, respectively, are found in the internal monolayer of eukaryotic plasma membrane, and participate in the regulation of several cellular processes, such as cell signaling and membrane transport (Kf de Campos and Schaaf, 2017). We show that the presence of all investigated anionic lipids causes a decrease in the pore edge tension and, consequently, in membrane stability.

## 2 Methods

PoET is an open-source software with a simple graphical user interface (GUI) that processes raw GUV image sequences in order to automatically measure pore diameter over time and to calculate pore edge tension. In the following subsection, we thoroughly describe how image processing is done and point out PoET features. Subsequently, we describe the experimental methods used to validate the software.

### 2.1 General features of the software

The software was developed in Python 3.6.8 and its GUI was built with WxPython 4.0.6. The general flowchart of PoET is shown in Figure 1 and the steps represented by capital letters in the figure are referenced along the text in parenthesis. At first, (A) the raw GUV Tagged Image File Format image sequences have their background subtracted by the sliding paraboloid algorithm [based on the rolling ball method (Sternberg, 1983)] in an external image processing software, like ImageJ (Schneider *et al.*, 2012). This first step is the only one done externally to PoET and it is not mandatory, but we recommend it as a guarantee to normalize images generated at different microscopy systems (see Supplementary Fig. S1 for a comparison between analysis with and without background subtraction). From this point onward, all procedures are done by PoET. For proper analysis, the images should satisfy a few requirements: (i) the GUV center should be well aligned with the center of the image (at the least the image center should fall inside the GUV image), which is important for the software to be able to identify left and right sides of the GUV; (ii) the pore should be in the bottom of the image; and (iii) the image sequence should contain a few images with the GUV fully closed, both before pore opening and after pore closure, which is necessary to measure GUV diameter and assess potential changes in size. All these requirements are easily achieved within an optional pre-processing interface of PoET, launched right after the image sequences are loaded on PoET and represented in Figure 1 by the corresponding roman numbers in Editing steps.

This pre-processing interface has basically three features: (i) cropping, (ii) 90° rotation and (iii) slicing in time. Cropping allows the user to manually crop the image sequence by either drawing a rectangle over the displayed image or to accept an automatically suggested area. Most of the image area (at least 70%) should be occupied by the vesicle, which also ensures that image center falls inside the vesicle, but, during the experiment, the vesicle may drift, especially after the application of the electroporating pulse. Therefore, the cropping tool estimates and displays a suggested cropping area that guarantees that the GUV will remain within the image frame throughout the whole image sequence. As illustrated in Supplementary Figure S2, the suggested area is produced by the following sequence of processes: generate the image maximum intensity projection using the whole image sequence, apply isodata threshold (Ridler and Calvard, 1978), label image elements, calculate the biggest element bounding box (a rectangle area that fully comprises this element) and expand the bounding box width and height by 20%, limited by the image frame. This ensures that requirement (i) for centering the GUV in the image is fulfilled and it works whether background was subtracted or not. The generated area and its center are displayed over the image sequence and the user may choose to readily apply it. The 90° rotation allows analysis of pores that originally face the top or the sides by rotating the entire image sequence within the program. This simple step is enough to fulfill requirement (ii) for localizing the pore at the image bottom. The next PoET feature, slicing in time, can be used to remove unnecessary extra frames in the beginning or end to decrease image analysis execution time. However, as mentioned earlier in requirement (iii), it is essential to leave a few frames displaying the GUV fully closed. The frame rate (in units of frames per second), microscope resolution (microns per pixel) and viscosity (in units of 10^−3^ Pa.s) are provided through the main interface.

Once an image sequence is loaded, (B) a Sobel filter (Parker, 2011) is applied to highlight the membrane; this accentuates image intensity gradient and facilitates membrane detection. Then, (C) isodata thresholding (Ridler and Calvard, 1978) is applied to generate binary images where the inner circular contour represents the GUV membrane, while the other elements represent the external halo and some undesirable specks (resulting e.g. from dirt in the optics, potential floating membranous structures, spurious hallo perturbation, etc.). It is important to point out here that, depending on the image, the external halo element may be absent in the binary image, but this does not affect the analysis. The image sequence is cropped in half its height (D) to focus the analysis only at the bottom part where the pore to be analyzed should be located. Then, a reference GUV membrane area is defined from the first image (the inner contour in Fig. 1 after step D) and specks are excluded by removing elements whose area is smaller than a percentage of this reference area, called threshold area. This threshold area can be modulated by the user through a sensitivity parameter, whose value changes the percentage from 0% to 15% (higher sensitivities tend to leave more elements in the images, see Supplementary Fig. S3 for more details). This range was established as a safe limit to avoid erasing the membrane itself. This strategy ensured that the same speck removal efficiency could be achieved regardless of the image resolution because the reference is the membrane area itself.

After that, (E) a custom labeling method is performed on the remaining elements by searching for white pixels from the top center of the image to each side of the image (see an example in Supplementary Fig. S4). This guarantees the identification of the inner contour rather than the external halo and highlights the importance of having removed potential specks inside the GUV that could mislead membrane element identification. If the detected elements are the same, it means the pore is closed, but if the elements have different labels, this means that the membrane contour is discontinuous, i.e. there is an open pore. Next step (F) consists of measuring the pore size (2*r*), basically by calculating the Euclidean distance between the edges of the pore (see Supplementary Fig. S5 for details).

PoET also measures GUV diameter (*2R*) automatically, both before pulse application and after the pore is closed: (G) from the thresholded image sequence, it takes the first and last images, which display the vesicle fully closed, (H) removes small particles (analogous to speck removal in step D) and labels the inner closed element (shown as a red contour after step H in Fig. 1); this element interior is filled and this filled area is bounded (blue circle after step H in Fig. 1). GUV diameter is calculated as the diameter of a circle with the same area as this filled region. This procedure can be followed with more details in Supplementary Figure S6. Equation (1) can only be employed for γ determination if *R* is assumed roughly constant during pore closure. PoET compares GUV radii before and after electric pulse application and GUVs with radii deviation larger than 5% (Portet and Dimova, 2010) are recommended to be discarded from the analysis. After both *R* and *r* are obtained, the term in the left hand side of Equation (1) is computed and (I) the pore dynamics graphic is plotted. Now the third stage of the pore dynamics corresponding to slow and linear pore closure must be identified. The user is prompted to manually select the linear region (J), which is fairly evident in most cases (a typical example is the graphic after step I in Fig. 1). Alternatively, an automated procedure for linear region detection is performed to guide the user (see Supplementary Fig. S7). Finally, PoET performs linear regression on the selected interval of data and (K) calculates the edge tension γ by resolving the coefficient $2\gamma /\left(3\pi \eta \right)$ in Equation (1). In all graphs of this work, the vesicle and pore radius are in units of µm and the time is in units of ms.

## 3 Software validation and applicability

### 3.1 Graphical user interface

**Fig. 2.**
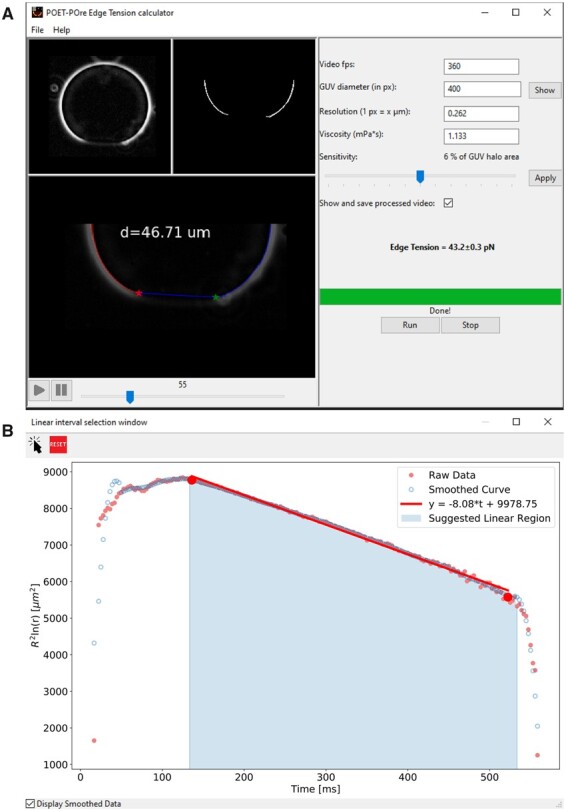
GUI of PoET. (**A**) Main window exhibiting a processed video in the bottom panel. (**B**) Pore dynamics graph of the raw (dark pink solid circles) and smoothed data (blue open circles), suggested linear region (light blue area) and a linear fit (red line) to the slow pore closure stage used for pore edge tension calculation.

PoET offers a friendly GUI, Figure 2, that is explained in detail in the user manual, which we provide together with the software, as well as in a Supplementary Video (see Supplementary Movie S1). This GUI enables the user to load image sequences of any size and bit depth (8- and 16-bit) in single ‘.tif’ format, which is typically obtained from any microscopy system or converted to from any other format (see the user manual for instructions on how to create single image sequence files with imageJ). After loading an image sequence, the user is given the option to edit it before analysis. The aim of the editing interface is to allow users to perform all the editing operations (image rotation, image cropping and video slicing) that are generally necessary before analysis within the software itself, without the need to use external image editing tools.

After the editing step, the image sequence is loaded into the software main interface, where it is possible to follow its original version, Figure 2A, upper left panel, as well as the processed one, Figure 2A, upper right panel. On the right side of the GUI, the user must enter the required parameters: acquisition rate, microscope resolution and medium viscosity. GUV diameters before pore opening and after pore closure are automatically measured when the image sequence is loaded. If the diameter variation is higher than 5%, a warning message pops up so the user may consider whether or not to discard the data. Aside from that, the final GUV diameter value displayed in the main interface and used to generate the pore dynamics graphic is the one measured from the last image, but can be manually edited by the user in case the measurement fails (e.g. if the GUV is not fully closed in the last frame). The sensitivity bar allows the user to select the elements to be analyzed according to their areas (as explained in Section 2.1 and Supplementary Fig. S3) and can be adjusted to guarantee that both side elements are visualized in the pre-processed video before pore diameter measurements. The user can choose, by means of a checkbox, either a complete analysis mode, where the processed image sequence will be displayed in PoET main interface (Fig. 2A, bottom panel, left side) and saved on the computer (see Supplementary Movie S2), or a fast mode, where the processed video is neither shown nor saved. The extra time for the complete analysis mode becomes noticeable only for long sequences (>3000 frames): complete mode analysis takes ∼68 ms per frame of typical size 370 × 370 px whereas fast mode takes around 13 ms per frame in a common laptop with 1.80 GHz and 16 GB RAM. In both modes, once the processing is done, a second window pops up displaying the pore dynamics graphic (Fig. 2B) with the original data (dark pink solid circles) and the smoothed version (blue open circles). The selection tool above the graphic should be used to mark two points that define the curve linear region (red dots in Fig. 2B). The graphic and a text file containing all pore sizes (µm) versus time (ms) are saved on the disk in an output folder created at the corresponding image sequence address.

A well-designed GUI makes the analysis intuitive and enables the user to interact with the software with clarity and precision. In our opinion, together with the saved time from laborious manual analysis, this is one of the major benefits of PoET. Furthermore, the user is not required to perform pre-processing of the video, nor have programming knowledge and in addition, is aided by the several error detection routines. PoET is available for Windows; the software, its code, the user manual and a reference GUV image sequence are freely available at https://dx.doi.org/10.17617/3.7h.

However, PoET has some limitations: first, the user is required to perform the image sequence background subtraction in an external software. Pyhton programming language currently lacks a routine for background subtraction that is both fast and reliable: the implementation of equivalent packages to ‘ImageJ Subtract Background’ routine have greatly increased video processing time, so we decided to leave this part as an optional, although recommended, external pre-processing step. Second, PoET is able to track only one pore at a time, which means that, if present, two smaller pores merging into a larger one are mistakenly considered as a single large pore from the beginning (see Supplementary Fig. S8B), thus, the pore opening stage is not correctly tracked. However, this does not influence the assessment of the pore edge tension. Furthermore, the model assumes constant vesicle radius during pore closure. In fact, an estimation of GUV and pore radii measurements from a synthetic image stack generated with a macro in ImageJ (see example and macro provided at https://dx.doi.org/10.17617/3.7h) indicates an overall reduction of 3% (GUV radius) and on average 11% (pore radius) when compared to the ground truth. Synthetic images mimic, although not completely, the real experiments. Thus, we also expect PoET to report smaller values than real ones. However, this would not affect pore edge tension calculation because the pore dynamics trend, namely the time dependence of $R^{2}\ln\left(r\right)$, is preserved, resulting in similar first derivative of the linear stage. The current limitations of PoET motivate improvements in future versions, like development of faster python-based algorithms for background subtraction and multiple pore tracking with characterization of other pore dynamics stages.

### 3.2. Software validation

#### 3.2.1 Comparison between manual and automated analysis

**Fig. 3.**
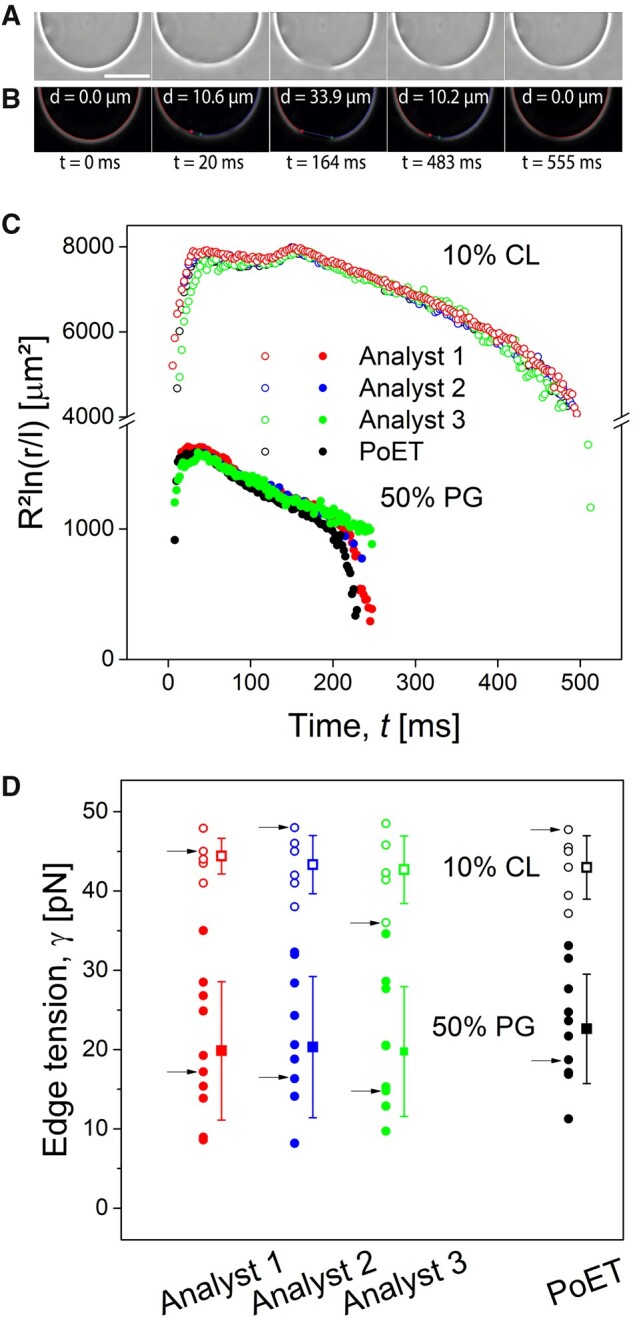
Comparison of manual and automated analysis. (**A**) Representative phase-contrast image sequence (360 frames per second) of a 10% CL GUV during application of an electroporating DC pulse (3 kV/cm, 150 µs). Scale bar corresponds to 50 µm. (**B**) Post-processed images obtained after analysis with PoET. Pore diameter, *d*, is indicated in each image. (**C**) Representative pore dynamics traces of 10% CL and 50% PG GUVs obtained manually by three analysts (blue, green and red circles) and automatically by PoET (black circles). (**D**) Edge tension values (individual measurements on different vesicles are displayed as circles; corresponding standard deviations are shown as error bars, open symbols show data from GUVs containing 10% CL and solid symbols relate to 50% PG) calculated from pore dynamics traces generated manually by three different analysts and automatically by PoET. Each arrow in (D) points to the edge tension value obtained from the matching color and style curve in (C).

The efficiency of our software was evaluated using 16 image sequences (each containing between 94 and 420 images), 6 of which were obtained with PC GUVs containing 10 mol% CL, and 10 of them, containing 50 mol% PG. Details on the GUV formation and observation protocol, as well as for manual image analysis are described in Supplementary Text S1. Three analysts manually measured pore diameters along those sequences (representative snapshots of the sequences are shown in Fig. 3A). Fit to the linear region of pore closure was obtained with Origin 8.0, from where the analysts acquired the edge tension in each case. Automatic analysis was performed on the same sequences with their backgrounds previously subtracted (post-processed images for the same GUV after analysis with PoET are shown in Fig. 3B) on a common laptop with 1.80 GHz and 16 GB RAM.

Figure 3C and D shows the comparison among manual measurements and PoET. The software was able to accurately detect and measure pore diameters in the GUVs and there were no striking differences among pore dynamics obtained by the three analysts and PoET (Fig. 3C). Thus, it is natural to expect that the edge tension obtained for individual GUVs would be consistent between measurements by analysts and PoET. This explains the absence of significant statistical difference for the average edge tension values, both for 10 mol% CL and 50 mol% PG groups (Fig. 3D) (*P* = 0.86 and *P* = 0.85, respectively, Bonferroni post-test after monofactorial analysis of variance).

Regarding the analysis time, the analysts spent, on average, 110 min analyzing the 16 videos. The employment of PoET greatly reduced whole data analysis time to 4 min (in the complete mode analysis).

We have observed that the accuracy of pore size measurement is directly dependent on image quality. We found that, in cases where the GUVs and opened pores were not well focused the manual results were dissimilar from each other and from PoET results, even with background removal as an attempt to mitigate possible quality degradation (Supplementary Fig. S8C). This confirms that inadequately focused GUVs pose a challenge to both automatic and manual pore characterization. Therefore, only GUV images whose pore is well focused at the equatorial plane and far from other artifacts should be used for edge tension measurements.

#### 3.2.2 Edge tension measurements of GUV membranes with different composition and amount of charge

**Fig. 4.**
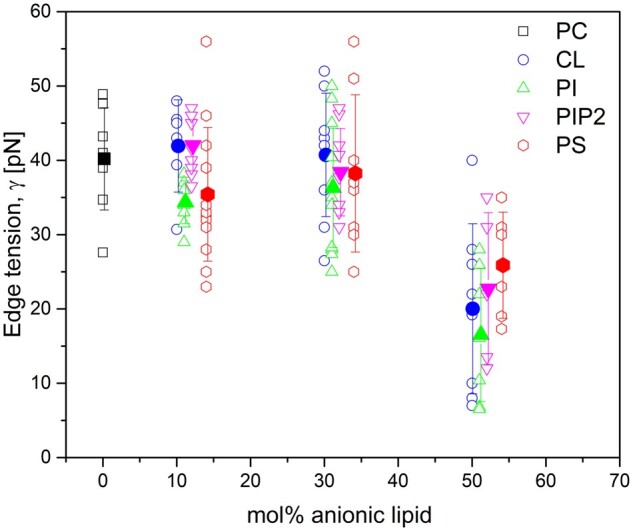
Values of edge tension obtained for membranes made of pure PC (black squares), PC: CL (blue circles), PC: PI (green triangles), PC: PIP2 (magenta inverted triangles) and PC: PS (red diamonds). Each point (open symbols) corresponds to one measurement on a single GUV, and solid symbols show the mean values with standard deviation (vertical bars) for each condition studied.

We validated and demonstrated the suitability of PoET for edge tension measurements by comparing the values for GUVs of different lipid compositions and fractions of negatively charged lipids. Figure 4 shows all the edge tension values measured, where each point represents a measurement on a single GUV, and filled symbols represent each group mean value with error bars being standard deviations, respectively. Table 1 shows all mean pore edge tension values obtained for all GUV compositions in this work and also in a previous work with PC: PG membranes. The values obtained for pure PC bilayers (40.2 ± 6.8 pN) are in accordance with previously reported results [40 ± 12 pN (Mattei *et al.*, 2017), 50 ± 10 pN (Casadei *et al.*, 2018) and 39 ± 5 pN (Lira *et al.*, 2021)] obtained with the same method (Portet and Dimova, 2010). The addition of 10 and 30 mol% of any of the studied anionic lipids (CL, PI, PIP2 or PS) to PC GUVs did not cause variations in pore edge tension, in agreement with results reported in our previous work with GUVs containing the same fractions of PG (Lira *et al.*, 2021). Significant statistical difference was only noticed for membranes containing 50 mol% anionic lipid (*P* < 0.05 for all cases compared to pure PC, monofactorial analysis of variance with Bonferroni post-test). This increased fraction of the anionic lipid caused an approximate 2-fold reduction in pore edge tension, regardless of which lipid was used. The overall decrease in edge tension for charged membranes agrees with previous data obtained with other methods, even though with different absolute values (Karal *et al.*, 2015, 2020; Levadny *et al.*, 2013).

**Table 1.** Edge tension values obtained on GUVs made of pure PC (0 mol% anionic lipid) and with 10, 30 and 50 mol% of anionic lipid.

Anionic lipid	0 mol% (pN)	10 mol% (pN)	30 mol% (pN)	50 mol% (pN)
PG^a^	40.2 ± 6.8	40.8 ± 5.1	39.0 ± 10.0	23.4 ± 6.0
PS		35.4 ± 9.0	38.3 ± 10.6	25.9 ± 7.1
PI		34.3 ± 2.9	36.3 ± 8.0	16.5 ± 8.9
CL		41.9 ± 6.2	40.7 ± 8.3	20.0 ± 11.5
PIP2		42.0 ± 3.4	38.4 ± 5.9	22.7 ± 10.2

*Note*: The data obtained with 50 mol% is the only condition with significant statistical difference between that group and pure PC (*P* <0.05, Bonferroni post-test after monofactorial analysis of variance).

a Data from Lira *et al.* (2021).

Our results for the membrane edge tension reinforce the idea that PI and PS are electrostatically similar to PG (Langner *et al.*, 1990), which we also confirmed by means of zeta potential measurements on small unilamellar vesicles (LUVs), shown in Supplementary Table S1, prepared with the same lipid stock solutions as those used for GUVs preparation. One could expect a reduction in pore edge tension for lower molar fractions of CL, since it exhibits double the charge of the other lipids. However, one should keep in mind that the area per lipid of CL is also almost twice as large as that of the other lipids (Pan *et al.*, 2015), therefore, the surface charge density does not differ much from that of the other lipids. Thereby, when comparing the surface charge density of membranes containing 10 and 30 mol% CL with that of membranes containing 50 mol% PG, whose surface charge density is known to cause a reduction in pore edge tension (Lira *et al.*, 2021), we observed that these values are much lower and that in fact they should not cause changes in pore edge tension. Yet, the surface charge density of membranes containing 50 mol% CL is slightly higher than that observed for 50 mol% PG membranes, which is reflected in the equal reduction in pore edge tension and also in the zeta potential measurement. With respect to PIP2, in fact, we expected to see a reduction in pore edge tension for compositions containing <50 mol%. Previous studies have reported that the electrostatic properties of PIP2 are quite different from those of other lipids (Langner *et al.*, 1990). Furthermore, due to its high electrostatic charge and high propensity to oxidation, having a high CMC (of the order of µM compared to nM for the other lipids), PIP2 can desorb from the outer monolayer of the membrane (Carvalho *et al.*, 2008; Shukla *et al.*, 2019). Thus, it is likely that our GUVs did not contain the expected molar fractions of PIP2 and that, in fact, the results obtained here reflect the behavior of membranes with lower fractions of PIP2. Finally, one could expect a slight discrepancy between the starting lipid mixture and the composition of the external GUV leaflet as demonstrated for electroformed vesicles containing 20% PG (Steinkühler *et al.*, 2018), which is not expected to be present in the LUVs used for measuring the zeta potential.

## 4 Conclusions

In this article, we presented the free and open-source software PoET for automated pore edge tension calculation in GUVs. PoET has proven to be able to accurately detect pore resealing dynamics enabling the correct determination of pore edge tension. We showed that results with our tool correspond well to those obtained manually, with the benefit of an ∼30-fold reduction of analysis time. This allowed us to examine a large population of GUVs composed of different lipid mixtures and achieve good data statistics. Besides that, we believe that automating analysis with PoET could standardize the way of measuring pore edge tension, avoiding discrepancies between users and found in the literature.

We studied poration of GUV membranes of different lipid compositions and surface charge to validate and show the applicability of PoET. Our results showed that membranes containing a high fraction of anionic lipids (50 mol%) exhibit reduced pore edge tension, and that this effect, which is associated with a decrease in the stability of these membranes, is not specific to lipid type (molecular architecture and charge in the polar head group), but rather to the surface charge density in the membrane.

We demonstrated the applicability of the PoET software and the pore edge tension analysis to membranes where the pores are caused by electric fields. However, as long as the pores are well visible in the equatorial section of a GUV, the way the pores are generated will not matter. We believe that the software could be used by researchers investigating pore formation caused by ultrasound (Newman and Bettinger, 2007), light (Sandre *et al.*, 1999), osmotic swelling (Shibly, S. U. A. *et al.*, 2016), and peptide interaction (Lee *et al.*, 2008), suggesting the wide applicability of the method.

## Supplementary Material

vbab037_Supplementary_Data

## References

[vbab037-B1] Alvarez-Erviti L. et al (2011) Delivery of siRNA to the mouse brain by systemic injection of targeted exosomes. Nat. Biotechnol., 29, 341–345. 21423189 10.1038/nbt.1807

[vbab037-B2] Baumgart T. et al (2003) Imaging coexisting fluid domains in biomembrane models coupling curvature and line tension. Nature, 425, 821–824. 14574408 10.1038/nature02013

[vbab037-B3] Belehradek M. et al (1993) Electrochemotherapy, a new antitumor treatment. First clinical phase I‐II trial. Cancer, 72, 3694–3700. 7504576 10.1002/1097-0142(19931215)72:12<3694::aid-cncr2820721222>3.0.co;2-2

[vbab037-B4] Bevers E. et al (1996) Regulatory mechanisms in maintenance and modulation of transmembrane lipid asymmetry: pathophysiological implications. Lupus, 5, 480–487. 8902787 10.1177/096120339600500531

[vbab037-B5] Brochard-Wyart F. et al (2000) Transient pores in stretched vesicles: role of leak-out. Phys. A Stat. Mech. Appl., 278, 32–51.

[vbab037-B6] Carvalho K. et al (2008) Giant unilamellar vesicles containing phosphatidylinositol(4,5) bisphosphate: characterization and functionality. Biophys. J., 95, 4348–4360. 18502807 10.1529/biophysj.107.126912 PMC2567945

[vbab037-B7] Casadei B.R. et al (2018) Bending modulus and edge tension of giant unilamellar vesicles (GUVS) composed of lipid extracts from erythrocytes membranes. Biophys. J., 114, 94a.

[vbab037-B8] Chiabrera A. et al (1985) Interactions between Electromagnetic Fields and Cells. Plenum Press, New York, NY.

[vbab037-B9] Dietrich C. et al (2001) Lipid rafts reconstituted in model membranes. Biophys. J., 80, 1417–1428. 11222302 10.1016/S0006-3495(01)76114-0 PMC1301333

[vbab037-B10] Dimova R. (2019) Giant vesicles and their use in assays for assessing membrane phase state, curvature, mechanics, and electrical properties. Annu. Rev. Biophys., 48, 93–119. 30811220 10.1146/annurev-biophys-052118-115342

[vbab037-B11] Dimova R. , Marques C. (2019) The Giant Vesicle Book. Taylor & Francis Group, CRC Press, Boca Raton, FL.

[vbab037-B12] Dimova R. et al (1999) Falling ball viscosimetry of giant vesicle membranes: finite-size effects. Eur. Phys. J. B, 12, 589–598.

[vbab037-B13] Dimova R. et al (2007) Giant vesicles in electric fields. Soft Matter, 3, 817–827. 32900072 10.1039/b703580b

[vbab037-B14] Dimova R. et al (2009) Vesicles in electric fields: some novel aspects of membrane behavior. Soft Matter, 5, 3201–3212.

[vbab037-B15] Döbereiner H.G. et al (2003) Advanced flicker spectroscopy of fluid membranes. Phys. Rev. Lett., 91, 048301. 12906698 10.1103/PhysRevLett.91.048301

[vbab037-B16] Edhemovic I. et al (2011) Electrochemotherapy: a new technological approach in treatment of metastases in the liver. Technol. Cancer Res. Treat., 10, 475–485. 21895032 10.7785/tcrt.2012.500224 PMC4527414

[vbab037-B17] Escoffre J.M. et al (2009) What is (still not) known of the mechanism by which electroporation mediates gene transfer and expression in cells and tissues. Mol. Biotechnol., 41, 286–295. 19016008 10.1007/s12033-008-9121-0

[vbab037-B18] Fadok V.A. et al (1998) The role of phosphatidylserine in recognition of apoptotic cells by phagocytes. Cell Death Differ., 5, 551–562. 10200509 10.1038/sj.cdd.4400404

[vbab037-B19] Faizi H.A. et al (2020) Fluctuation spectroscopy of giant unilamellar vesicles using confocal and phase contrast microscopy. Soft Matter, 16, 8996–9001. 10.1039/d0sm00943a 32966528

[vbab037-B20] Garten M. et al (2017) Whole-GUV patch-clamping. Proc. Natl. Acad. Sci. USA, 114, 328–333. 28003462 10.1073/pnas.1609142114 PMC5240670

[vbab037-B21] Heller L. , Heller R. (2010) Electroporation gene therapy preclinical and clinical trials for melanoma. Curr. Gene Ther., 10, 312–317. 20557286 10.2174/156652310791823489

[vbab037-B22] Henriksen J.R. , Ipsen J.H. (2004) Measurement of membrane elasticity by micro-pipette aspiration. Eur. Phys. J. E, 14, 149–167. 15254835 10.1140/epje/i2003-10146-y

[vbab037-B23] Honerkamp-Smith A.R. et al (2013) Membrane viscosity determined from shear-driven flow in giant vesicles. Phys. Rev. Lett., 111, 038103. 23909365 10.1103/PhysRevLett.111.038103

[vbab037-B24] Karal M.A.S. et al (2015) Electrostatic interaction effects on tension-induced pore formation in lipid membranes. Phys. Rev. E Stat. Nonlin. Soft Matter Phys., 92, 012708. 26274204 10.1103/PhysRevE.92.012708

[vbab037-B25] Karal M.A.S. et al (2019) Effects of electrically-induced constant tension on giant unilamellar vesicles using irreversible electroporation. Eur. Biophys. J., 48, 731–741. 31552440 10.1007/s00249-019-01398-9

[vbab037-B26] Karal M.A.S. et al (2020) Electrostatic effects on the electrical tension-induced irreversible pore formation in giant unilamellar vesicles. Chem. Phys. Lipids, 231, 104935. 32569600 10.1016/j.chemphyslip.2020.104935

[vbab037-B27] Keating A. , Toneguzzo F. (1990) Gene transfer by electroporation: a model for gene therapy. Prog. Clin. Biol. Res., 333, 491–498. 2308997

[vbab037-B28] Kf de Campos M. , Schaaf G. (2017) The regulation of cell polarity by lipid transfer proteins of the SEC14 family. Curr. Opin. Plant Biol., 40, 158–168. 29017091 10.1016/j.pbi.2017.09.007

[vbab037-B29] Kinosita K. et al (1988) Electroporation of cell membrane visualized under a pulsed-laser fluorescence microscope. Biophys. J., 53, 1015–1019. 3395657 10.1016/S0006-3495(88)83181-3 PMC1330281

[vbab037-B30] Kotnik T. et al (2015) Electroporation-based applications in biotechnology. Trends Biotechnol., 33, 480–488. 26116227 10.1016/j.tibtech.2015.06.002

[vbab037-B31] Langner M. et al (1990) Electrostatics of phosphoinositide bilayer membranes. Theoretical and experimental results. Biophys. J., 57, 335–349. 2156577 10.1016/S0006-3495(90)82535-2 PMC1280674

[vbab037-B32] Lee M.T. et al (2008) Mechanism and kinetics of pore formation in membranes by water-soluble amphipathic peptides. Proc. Natl. Acad. Sci. USA, 105, 5087–5092. 18375755 10.1073/pnas.0710625105 PMC2278198

[vbab037-B33] Levadny V. et al (2013) Rate constant of tension-induced pore formation in lipid membranes. Langmuir, 29, 3848–3852. 23472875 10.1021/la304662p

[vbab037-B34] Lira R.B. et al (2021) To close or to collapse: the role of charges on membrane stability upon pore formation. Adv. Sci., 8, 2004068. 10.1002/advs.202004068 PMC8188222 34105299

[vbab037-B35] Mattei B. et al (2017) Membrane permeabilization induced by Triton X-100: the role of membrane phase state and edge tension. Chem. Phys. Lipids, 202, 28–37. 27913102 10.1016/j.chemphyslip.2016.11.009

[vbab037-B37] Muralidharan A. et al (2021) Actin networks regulate the cell membrane permeability during electroporation. Biochim. Biophys. Acta Biomembr., 1863, 183468. 32882211 10.1016/j.bbamem.2020.183468

[vbab037-B38] Neumann E. et al (1982) Gene transfer into mouse lyoma cells by electroporation in high electric fields. EMBO J., 1, 841–845. 6329708 10.1002/j.1460-2075.1982.tb01257.x PMC553119

[vbab037-B39] Newman C.M.H. , Bettinger T. (2007) Gene therapy progress and prospects: ultrasound for gene transfer. Gene Ther., 14, 465–475. 17339881 10.1038/sj.gt.3302925

[vbab037-B40] Pan J. et al (2015) Structural and mechanical properties of cardiolipin lipid bilayers determined using neutron spin echo, small angle neutron and X-ray scattering, and molecular dynamics simulations. Soft Matter, 11, 130–138. 25369786 10.1039/c4sm02227k

[vbab037-B41] Parker J.R. (2011) Algorithms for Image Processing and Computer Vision. 2nd edn. John Wiley & Sons, Inc., New York.

[vbab037-B42] Perrier D.L. (2018) Electroporation of biomimetic vesicles. TU Delft Univ., 91, 165.

[vbab037-B43] Perrier D.L. et al (2017) Lipid vesicles in pulsed electric fields: fundamental principles of the membrane response and its biomedical applications. Adv. Colloid Interface Sci., 249, 248–271. 28499600 10.1016/j.cis.2017.04.016

[vbab037-B44] Portet T. , Dimova R. (2010) A new method for measuring edge tensions and stability of lipid bilayers: effect of membrane composition. Biophys. J., 99, 3264–3273. 21081074 10.1016/j.bpj.2010.09.032 PMC2980741

[vbab037-B45] Potter H. , Heller R. (2018) Transfection by electroporation. Curr. Protoc. Mol. Biol., 121, 9.3.1.–9.3.13. 10.1002/cpmb.48 29337375

[vbab037-B46] Rawicz W. et al (2000) Effect of chain length and unsaturation on elasticity of lipid bilayers. Biophys. J., 79, 328–339. 10866959 10.1016/S0006-3495(00)76295-3 PMC1300937

[vbab037-B47] Ridler T.W. , Calvard S. (1978) Picture thresholding using an iterative selection method. IEEE Trans. Syst. Man Cybern., 8, 630–632.

[vbab037-B48] Riske K.A. , Dimova R. (2005) Electro-deformation and poration of giant vesicles viewed with high temporal resolution. Biophys. J., 88, 1143–1155. 15596488 10.1529/biophysj.104.050310 PMC1305119

[vbab037-B49] Riske K.A. et al (2009) Bursting of charged multicomponent vesicles subjected to electric pulses. Soft Matter, 5, 1983–1986.

[vbab037-B50] Ryham R. et al (2011) Aqueous viscosity is the primary source of friction in lipidic pore dynamics. Biophys. J., 101, 2929–2938. 22208191 10.1016/j.bpj.2011.11.009 PMC3244058

[vbab037-B51] Salipante P.F. et al (2012) Electrodeformation method for measuring the capacitance of bilayer membranes. Soft Matter, 8, 3810.

[vbab037-B52] Sandre O. et al (1999) Dynamics of transient pores in stretched vesicles. Proc. Natl. Acad. Sci. USA, 96, 10591–10596. 10485870 10.1073/pnas.96.19.10591 PMC17927

[vbab037-B54] Schneider C.A. et al (2012) NIH image to ImageJ: 25 years of image analysis. Nat. Methods, 9, 671–675. 22930834 10.1038/nmeth.2089 PMC5554542

[vbab037-B55] Schwille P. et al (1999) Fluorescence correlation spectroscopy with single-molecule sensitivity on cell and model membranes. Cytometry, 36, 176–182. 10404965 10.1002/(sici)1097-0320(19990701)36:3<176::aid-cyto5>3.0.co;2-f

[vbab037-B53] Shibly U.A.S. et al (2016) Experimental estimation of membrane tension induced by osmotic pressure. Biophys. J., 111, 2190–2201. 27851942 10.1016/j.bpj.2016.09.043 PMC5112947

[vbab037-B56] Shukla S. et al (2019) PIP2 reshapes membranes through asymmetric desorption. Biophys. J., 117, 962–974. 31445680 10.1016/j.bpj.2019.07.047 PMC6731468

[vbab037-B57] Steinkühler J. et al (2018) Charged giant unilamellar vesicles prepared by electroformation exhibit nanotubes and transbilayer lipid asymmetry. *Sci. Rep.*, **8**, 11838. 10.1038/s41598-018-30286-z PMC6081385 30087440

[vbab037-B58] Sternberg S.R. (1983) Biomedical image processing. Computer, 16, 22–34.

[vbab037-B59] Unsay J.D. et al (2013) Cardiolipin effects on membrane structure and dynamics. Langmuir, 29, 15878–15887. 23962277 10.1021/la402669z

[vbab037-B60] Walde P. et al (2010) Giant vesicles: preparations and applications. ChemBioChem, 11, 848–865. 20336703 10.1002/cbic.201000010

[vbab037-B61] Zhelev D.V. , Needham D. (1993) Tension-stabilized pores in giant vesicles: determination of pore size and pore line tension. BBA Biomembr., 1147, 89–104. 10.1016/0005-2736(93)90319-u 8466935

