# Supplementary material for: PoET: automated approach for measuring pore edge tension in giant unilamellar vesicles
Source: Bioinform Adv. 2021 Nov 23;1(1):vbab037. doi: 10.1093/bioadv/vbab037 (PMC9710609; doi:10.1093/bioadv/vbab037)
Supplement: vbab037_Supplementary_Data [file vbab037_supplementary_data.pdf]

Supplementary Data

PoET: automated measurement of pore edge tension in giant unilamellar vesicles

Fernanda S. C. Leomil[1,2], Marcelo Zoccoler[3], Rumiana Dimova[2*] and Karin A. Riske[1*]

[1] *Departamento de Biofísica, Universidade Federal de São Paulo, 043039-032, Brazil.*
[2] *Department of Theory and Biosystems, Max Planck Institute of Colloids and Interfaces, Potsdam 14424, Germany.*
[3] *DFG Cluster of Excellence "Physics of Life", Technical University of Dresden, Dresden 01307, Germany.*

∗ *E-mails for correspondence kariske@unifesp.br, rumiana.dimova@mpikg.mpg.de*

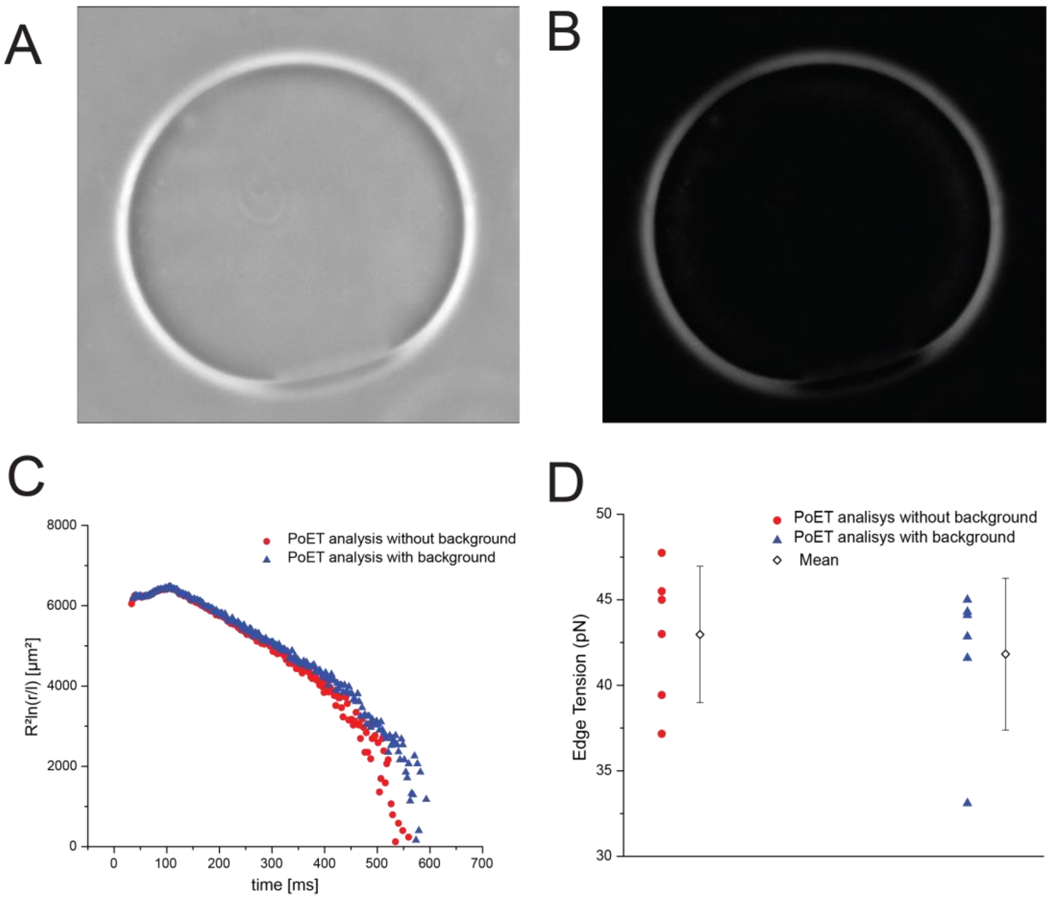

Figure S1. Comparison of pore measurements with and without background subtraction. (A) Raw image obtained from phase-contrast microscopy. (B) Same image after background removal with ImageJ (command 'Subtract Background' with sliding paraboloid and 50-pixel radius). (C) Traces of pore dynamics obtained after analysis with and without background subtraction for the same GUV. (D) Edge tension values (means and standard deviations shown in black) calculated from pore dynamics traces generated by PoET for POPC GUVs containing 10% CL.

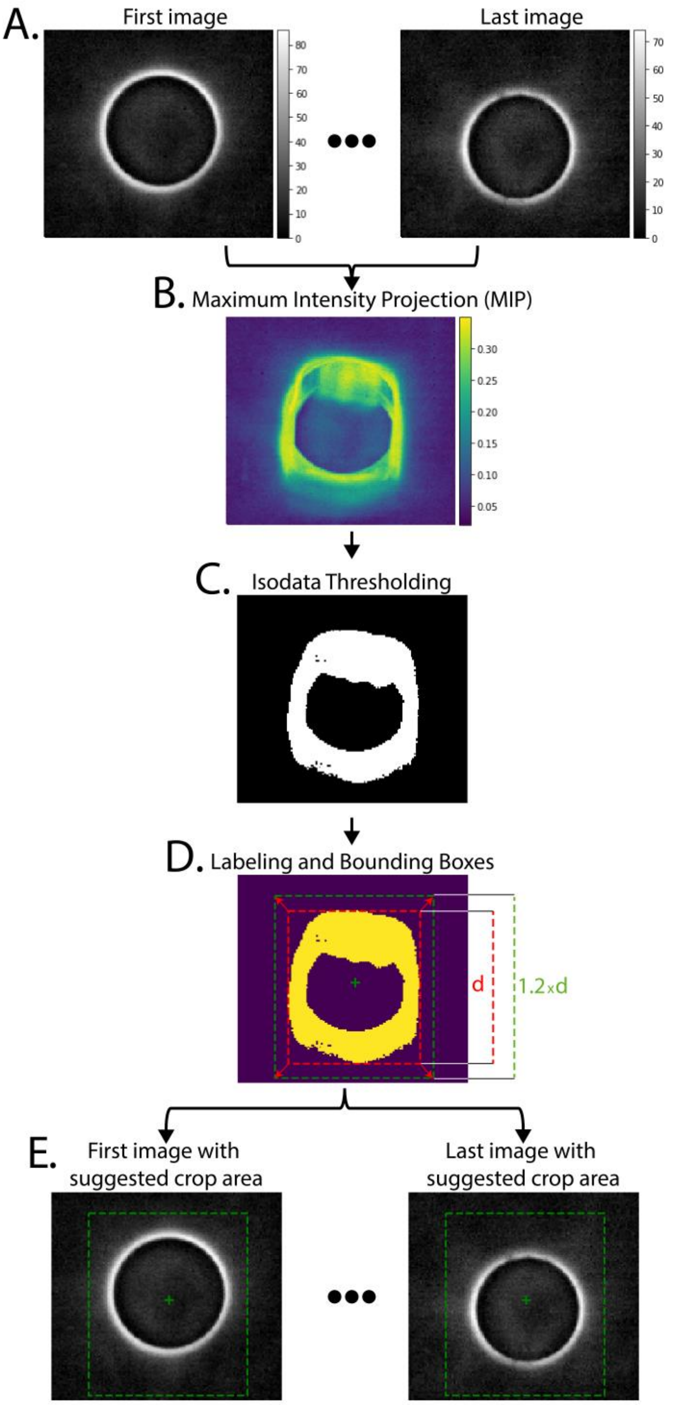

Figure S2. Automatic cropping area method: (A) Initial first and last images. (B) the maximal intensity projection (MIP) is generated from the whole image sequence. (C) Isodata thresholding is applied on the MIP image. (D) All elements in the image are labeled and the biggest element bounding box width and height are expanded by 20%. (E) The final bounding box is displayed over the images.

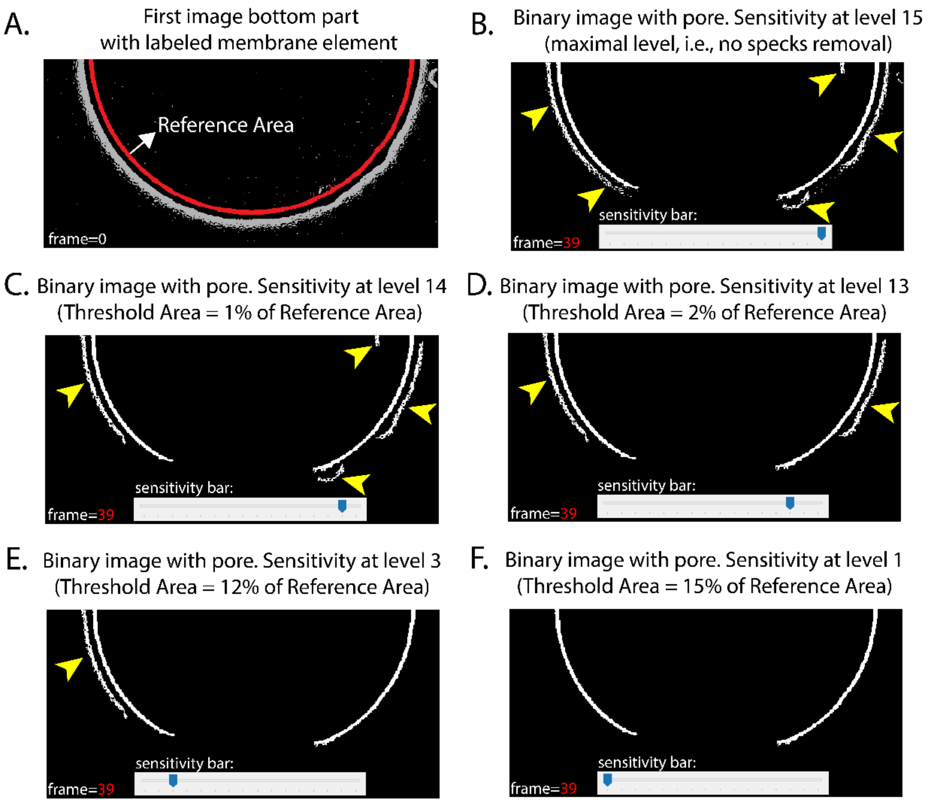

Figure S3. Illustration of varying the sensitivity bar in PoET. (A) First image bottom half with reference membrane area labeled in red. (B) Binary image with an open pore and sensitivity bar at maximal level, which means that no specks are removed (yellow arrow heads point to some specks). (C) Sensitivity bar at level 14 (elements whose area is smaller than 1% of membrane area are removed). (D) Sensitivity bar at level 13 (elements whose area is smaller than 2% of membrane area are removed). (E) Sensitivity bar at level 3 (elements whose area is smaller than 12% of membrane area are removed). (F) Sensitivity bar at level 1 (elements whose area is smaller than 15% of membrane area are removed). In this GUV example, there is no change from level 13 to 3.

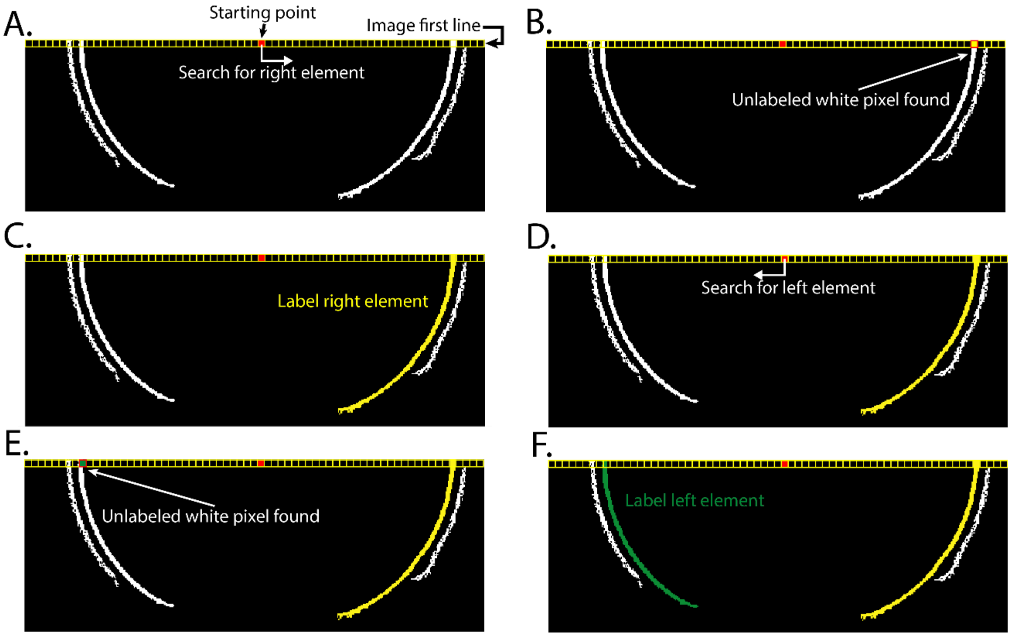

Figure S4. Custom labeling method to identify left and right inner elements of the membrane contour. (A) From the top center of the thresholded bottom image, a search is performed in the first line of pixels to the right. (B) The search stops when a white pixel is found. (C) The whole element is identified and labeled as the right element. (D) The same search is repeated, but now to the left. (E) The search stops again when a white pixel is found. (F) The corresponding element is labeled as the left one.

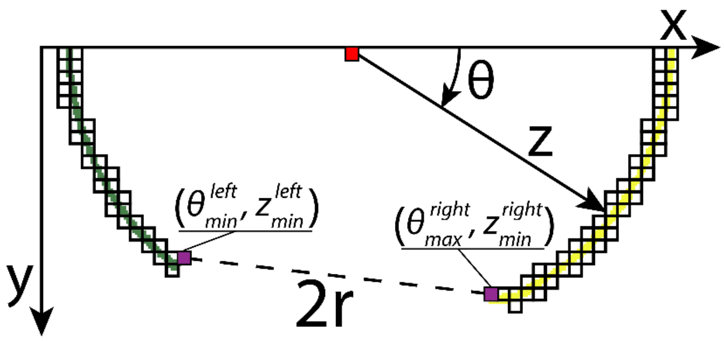

Figure S5. Pore size measurement diagram. The vesicle contour is represented with pixels of exaggerated size. The polar coordinates of the edges of the left and right elements (purple pixels) are found and the distance 2r is calculated. Both Cartesian (x,y) and polar (z,θ) coordinates are displayed with the membrane elements represented by exaggerated pixels. Each element extremity is found, which is done in three steps: (i) all pixel coordinates belonging to an element (yellow or green) are converted to polar coordinates with the origin of the polar coordinate system being the top center of the image (red pixel in Figure S5, top); (ii) for the right element (yellow), pixels with the largest θ coordinate are selected (θ_{max}^{right}) while for the left element (green), pixels with the smallest θ coordinate are selected (θ_{min}^{left}); (iii) on rare occasions where more than one pixel have the same θ, the one with smallest z is selected. The Euclidean distance between these edge pixels is calculated and computed as the pore diameter (2*r*).

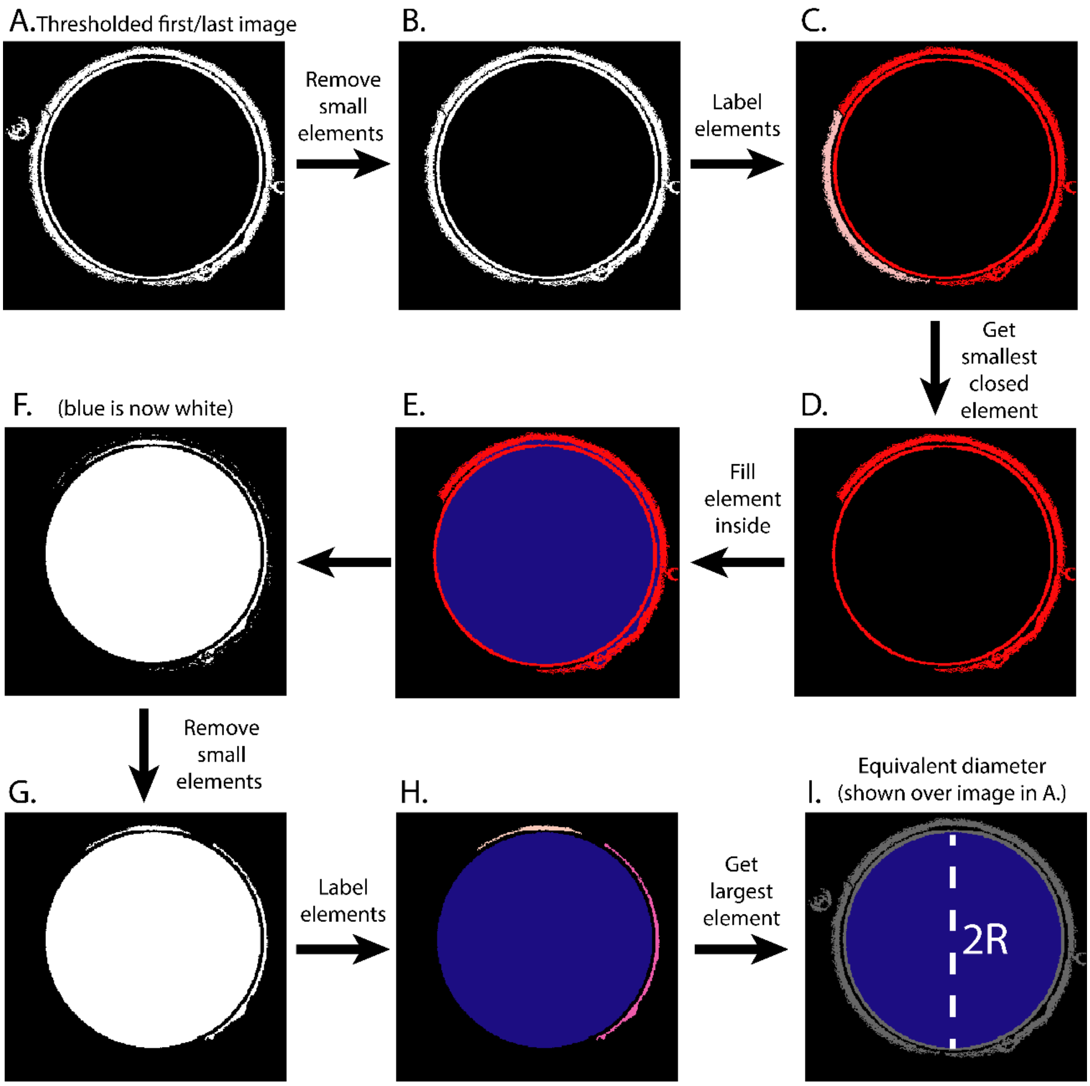

Figure S6. Method for automatic GUV diameter measurement. Binary first or last image (A) undergoes the procedure that removes elements smaller than threshold area (defined by the sensitivity bar), resulting in (B). Then, all connected elements are labeled (C) and just the smallest closed one is kept (D, red element). This element interior is filled (E, blue region) and this filled region is kept while the rest is removed (F, where blue region is now shown in white). Small elements are removed from the new image by the same criterion (G) and the remaining elements are labeled (H). Finally, the largest element is kept while the others are removed and its equivalent diameter is calculated and defined as the GUV diameter (I, final region shown in blue over image from A.).

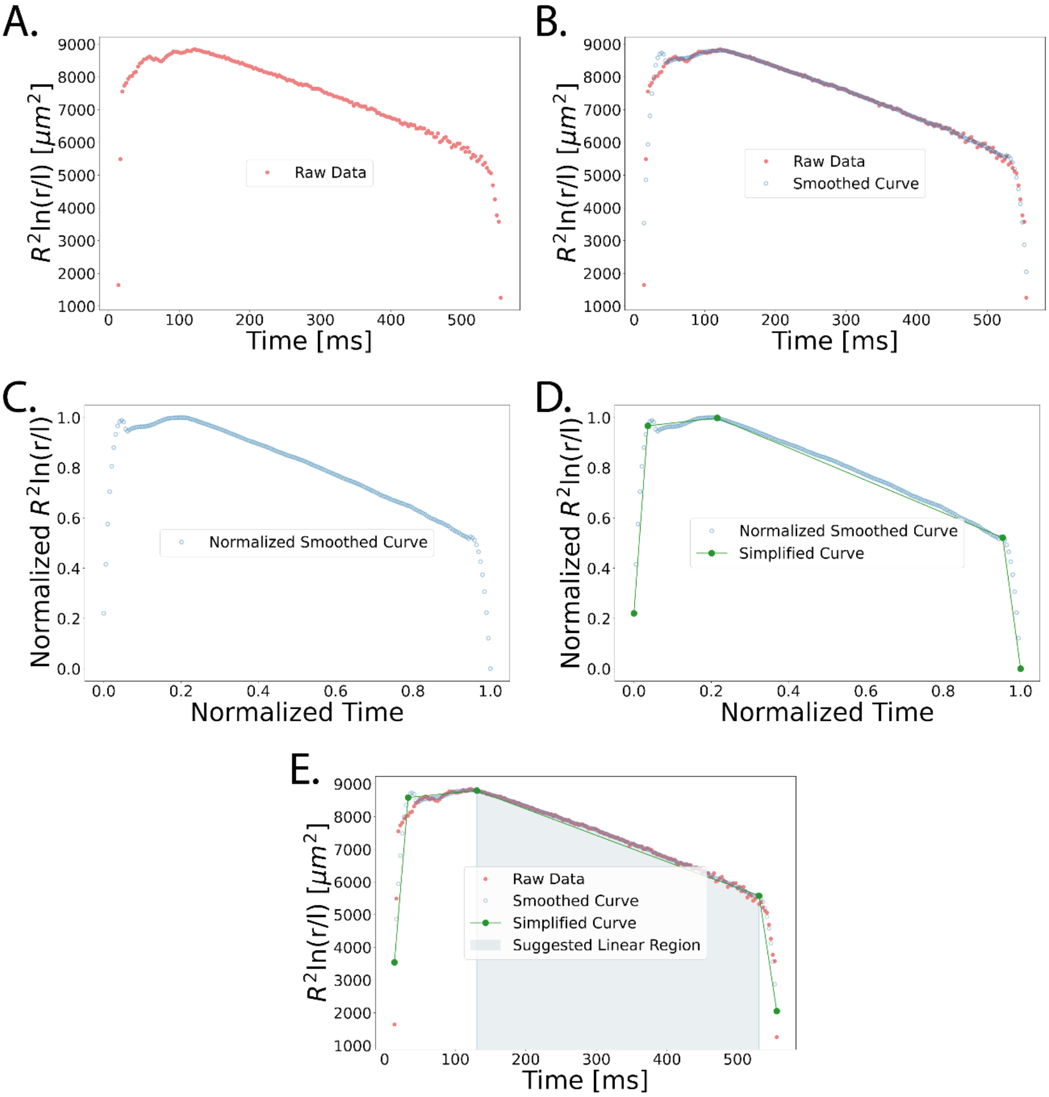

Figure S7. Method for automatic detection of largest linear region. (A) Raw data for pore dynamics (pink filled circles). (B) Smoothed data (blue open circles) by a Savitzky–Golay digital filter (window size = 10% data length, polynomial degree = 3). (C) Y-axis and X-axis are normalized by the respective maximal value in the data. (D) Simplified curve (green line and solid green circles) by applying Ramer-Douglas-Peucker algorithm (ε=0.1) to the normalized smoothed curve. (E) Identification of the largest line from the simplified curve (blue shaded region) as being the suggested linear region of pore dynamics graphic.

The automatic detection of the largest linear region in the pore dynamics graph of: (i) smoothing the curve by a Savitzky–Golay digital filter (Schafer, 2011) (window size equal to 10% of the amount of data points and third-degree polynomial), which is useful to reduce quantization noise; (ii) normalizing the curve y-axis; (iii) applying a Ramer-Douglas-Peucker algorithm (Ramer, 1972; Douglas and Peucker, 2011) (ε=0.1); and (iv) finding the largest line segment interval. The smoothed curve and the suggested region can be optionally displayed over raw data. The filter parameters were chosen empirically, but based on the fact that higher polynomial degrees and smaller windows allow more abrupt variations, making the filter ineffective, while larger windows risk deforming the curve by excessive smoothing. The ε parameter was also chosen empirically and its value means that any point whose distance is smaller than 0.1 from a previously identified line segment is excluded.

Text S1: Experimental methods for vesicle preparation, observation and image analysis

The phospholipids 1-palmitoyl-2-oleoyl-sn-glycero-3-phosphocholine (PC), 1',3'-bis[1,2-dioleoyl-sn-glycero-3-phospho]-glycerol (sodium salt) (CL), 1,2-dioleoyl-sn-glycero-3-phospho-L-serine (sodium salt) (PS), L-α-phosphatidylinositol (Soy) (sodium salt) (PI) and L-α-phosphatidylinositol-4,5-bisphosphate (Brain, Porcine) (ammonium salt) (PIP2) were purchased from Avanti Polar Lipids (Alabaster, AL). GUVs were prepared by the electroformation method(Angelova and Dimitrov, 1986) that basically consists of depositing the phospholipid mixture, dissolved in an organic solvent (chloroform), on the surfaces of two conductive glasses. After drying the films, the glasses were sealed with a Teflon spacer (2 mm thick) in between to form a chamber (of volume 2 mL) which was filled with a 0.2 M sucrose solution with 0.1 mM EDTA and connected to a function generator. The formation of vesicles was accelerated by the application of AC fields (1V, 10 Hz), and completed in approximately 1 hour. Before the experiments, GUVs were diluted ~10x in a 0.2 M glucose solution containing 0.1 mM EDTA (to eliminate possible impurities of divalent ions such as calcium) and 0.5 mM NaCl. The vesicle solution (900 µL) was placed in an observation chamber (Eppendorf, Hamburg, Germany) which consisted of two parallel platinum electrodes (92 µm in radius) and 500 µm apart. The chamber was connected to a Multiporator (Eppendorf) for DC electric pulse application (3 kV/cm, 150 µs). GUVs were observed in a Zeiss Axiovert 200 microscope (Jena, Germany) equipped with a sCMOS PCO.edge 4.2 digital camera (Kelheim, Germany) and with a 20x objective, NA 0.4. The acquisition rate was between 300 and 600 frames per second (fps). Pore dynamics was either automatically assessed with PoET, where for η we used 1.133×10^{-3} Pa.s (viscosity of the 0.2 M glucose solution (Portet and Dimova, 2010)), or by manually measuring pore size on the image sequences using ImageJ, for means of comparison. In the latter case, linear fits to the pore closure dynamics were done with Origin 8.0 (OriginLab Corp., Northampton, MA, USA).

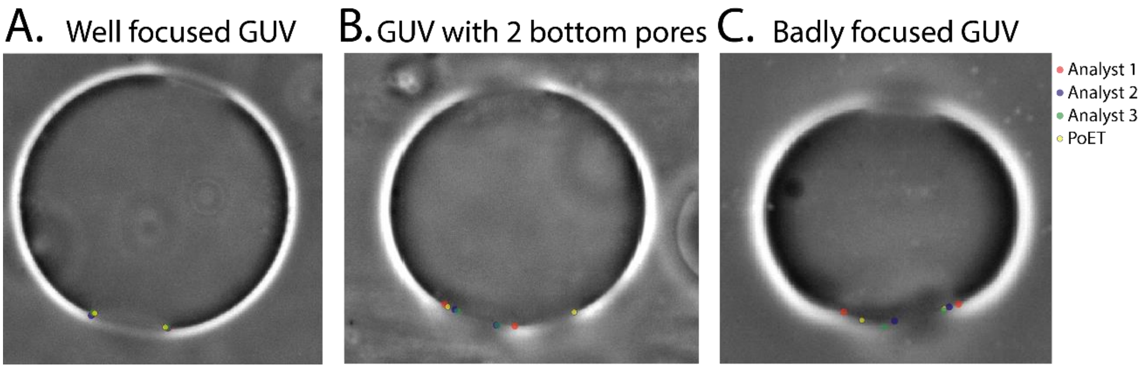

Figure S8. Examples of different data analyzed by three analysts and PoET. Each analyst was unaware of the others markings. (A) Well focused GUV image displaying a good match between analysts and PoET. (B) GUV with two pores in the bottom region demonstrating a limitation of PoET, which considers the two pores as a single bigger pore. (C) Badly focused GUV highlighting large measurement variation within analysts and also with PoET.

Table S1: Zeta potential measurements on LUVs of POPC with and without 50 mol% of anionic lipid.

Composition	Zeta Potential (mV)
POPC	-18.0±2.8
50 mol% PG	-37.1±2.0
50 mol% PS	-39.6±1.4
50 mol% PI	-40.7±2.1
50 mol% CL	-46.0±3.6
50 mol% PIP2	-47.2±1.0

Raw data and extra files

The program and its python code, the user manual, a set of raw data, supplementary files and the software license are available at https://dx.doi.org/10.17617/3.7h. This material is organized in different folders:

- The folder “Software” contains a zipped file named “PoET_folder” with the .exe file for the software and a folder called “icones” inside. These two have to be copied to the user’s computer, as explained in the user manual, so that the software can work properly.

- The folder “Code” contains the python code.

- The folder “Raw data for analysis” contains a GUV image sequence in .tif format and a text file with information to be used for pore edge tension calculation using PoET.

- The folder “User Manual & Raw Data” contains the user manual, that should be read before trying to open the .exe file, the image sequence that was used in this manual for the step-by-step example and a text file with information to be inserted in the software.

- The folder “Synthetic stack” contains an imageJ macro for synthetic GUV generation (.ijm file), the resulting image stack (.tif file) and an excel file with the ground truth and measured radii values.

- The folder “Supplementary files” contains two supplementary movies:

Movie S1: a tutorial on how to use PoET.

Movie S2: a video exhibiting simultaneously the original and post-processed image sequence with pore size tracking.

References

Angelova,M.I. and Dimitrov,D.S. (1986) Liposome electroformation. *Faraday Discuss. Chem. Soc.*, **81**, 303.

Douglas,D.H. and Peucker,T.K. (2011) Algorithms for the Reduction of the Number of Points Required to Represent a Digitized Line or its Caricature. *Class. Cartogr. Reflections Influ. Artic. from Cartogr.*, 15–28.

Portet,T. and Dimova,R. (2010) A new method for measuring edge tensions and stability of lipid bilayers: Effect of membrane composition. *Biophys. J.*

Ramer,U. (1972) An iterative procedure for the polygonal approximation of plane curves. *Comput. Graph. Image Process.*, **1**, 244–256.

Schafer,R.W. (2011) What is a savitzky-golay filter? *IEEE Signal Process. Mag.*, **28**, 111–117.
